# Editorial: Elbow injury in pediatric patients

**DOI:** 10.3389/fped.2023.1228234

**Published:** 2023-06-22

**Authors:** Tianjing Liu, Qiang Jie, Enbo Wang, Lianyong Li, Federico Canavese

**Affiliations:** ^1^ Department of Pediatric Orthopedics, Shengjing Hospital of China Medical University, Shenyang City, China; ^2^ Department of Pediatric Orthopedics, Xi’an Honghui Hospital, Xi'an, China; ^3^ Department of Pediatric Orthopedics, Lille University Center - Jeanne de Flandre Hospital, Lille, France; ^4^ Faculty of Medicine, Nord-de-France Lille University, Lille, France

**Keywords:** elbow, children, fracture, supracondylar, lateral condyle, ultrasound

**Editorial on the Research Topic** Elbow injury in pediatric patients

Fractures of the distal humerus in children and adolescents present a diagnostic and therapeutic challenge to the orthopedic surgeon. “Pity the young surgeon whose first case is a fracture around the elbow,” said Mercer Rang to emphasize the difficulties inherent in traumatic injuries to the child's elbow (1).

The distal end of the humerus is the second most common site of fracture in children, yet it is the most common indication for surgery. The distal end of the humerus, in addition to its anatomical complexity, is characterized by the presence of numerous ossification nuclei that appear at different times during skeletal maturation (2). These elements are responsible for the many uncertainties in the treatment of traumatic injuries that occur in this anatomical region, in addition to their scientific understanding.

A total of 12 articles and one commentary were selected after selective peer review for the Special Issue of *Elbow Injury in Pediatric Patients* to provide the latest diagnostic and therapeutic strategies for fractures, both common and rare, of the distal end of the humerus in children.

Despite their frequency, not all supracondylar humerus fractures (SHF) require surgical fixation. In particular, Coupal et al. investigated the optimal form of immobilization for the treatment of Gartland-type 1 SHFs and found that there was insufficient high-quality evidence to determine the best option. They reported that children treated with a cuff and collar had delayed return to normal daily life activities and experienced more pain than those treated with a posterior splint. However, no studies directly compared posterior splints with circumferential casts.

Qian et al. assessed the learning curve for successful reduction and fixation of SHFs and reported that 65 procedures are needed to master the surgical technique of closed reduction (CR) and percutaneous fixation; interestingly, they also pointed out that surgical experience significantly impacts the post-operative recovery of children with such injuries.

However, CR and percutaneous fixation are more difficult to achieve in children with an SHF presenting more than 14 days after the initial trauma. Liu et al. reported that CR with a minimally invasive technique followed by external fixation is a potential alternative to manage SHFs presenting 2 or more weeks after the initial trauma; the preliminary results are encouraging with satisfactory functional outcome and low complication rate.

Flexion-type SHFs are extremely challenging to treat due to their instability and rotation of the distal fragment. Sun et al. reported that flexion-type SHFs have a higher rate of ulnar nerve injury and are at a higher risk of open reduction, particularly when lateral displacement and rotation are present simultaneously.

Weng et al. compared the clinical and radiographic outcomes of CR versus open reduction in the treatment of severely displaced lateral condyle fractures (LCFs). They found that despite the relatively long learning curve, CR of severely displaced LCFs is challenging and successful reduction cannot always be achieved. They concluded that although CR of severely displaced LCFs has some advantages, including a smaller scar and lower rate of postoperative infection, open reduction and percutaneous fixation still remain the first-line treatment for such injuries. In their commentary, Rehm et al. highlighted that CR and percutaneous fixation of LCFs with >4 mm displacement are feasible in a significant proportion of cases with relatively good outcomes.

Magnetic Resonance Imaging (MRI) has already been shown to accurately diagnose LCFs and is a valuable tool to properly restore joint anatomy during surgery (3). The use of intraoperative ultrasound (US) to guide both the reduction and stabilization of fractures is steadily increasing in pediatric traumatology (4). Deng et al. reported a novel approach for the treatment of displaced LCFs. They advocated that LCFs should first be reduced by CR and fixed with two to three Kirschner wires (1.5–1.8 mm) inserted under intraoperative US guidance; interestingly, they pointed out that fragments with >4 mm displacement are easier to visualize with US. The reported technique reduces radiation exposure, has a relatively low complication rate, and provides a good functional outcome, even though the results are preliminary and from a single center (4). Similarly, Li et al. investigated the use of intraoperative US guidance in Elastic Stable Intramedullary Nailing (ESIN) for pediatric humeral shaft fractures. They found that US-guided CR and ESIN fixation decreased the risk of radial nerve injury. However, intraoperative US cannot completely replace the role of radiography in humeral fracture surgery, although it can significantly reduce radiation exposure.

Although many surgical techniques have been reported for the management of displaced intercondylar fractures (DIFs) of the humerus in children, there is no specific and accepted treatment protocol for such injuries. Shu et al. reported the results of CR, external fixation, and percutaneous pinning for the treatment of DIFs. They found that fracture stability and acceptable clinical and functional outcomes could be achieved in patients younger than 10 years of age. It has been reported that DIFs in children older than 10 years of age have a higher complication rate and poorer functional outcomes compared to younger children (5). Despite the satisfactory outcome reported by Shu et al. the treatment of such injuries remains challenging and yields unpredictable outcomes.

The group of Monteggia-equivalent fractures (MEFs) has grown steadily over the last 10 years and has complemented Bado's classic classification system. Su et al. evaluated the treatment and outcome of radial head and neck fractures associated with a fracture of the ulna, which is a very rare form of MEF. They recommended anatomic reduction and internal fixation of the ulna to restore its length, and CR and ESIN fixation of the radial neck fracture. If these principles are followed, and early rehabilitation is performed, the functional outcome is very satisfactory in most cases.

Distal forearm fractures have rarely been reported in association with Monteggia type III fractures. In their review of the literature, Gao et al. could only identify four cases of this particular association in children. They reported the case of a 9-year-old boy with a type III Monteggia fracture, ipsilateral forearm fracture, and concomitant radial nerve deficit. The patient underwent open reduction and internal fixation of the distal forearm and proximal ulna. The functional and radiologic outcome of the patient was good with full recovery of the radial nerve injury at 1-year follow-up.

The coronoid process of the ulna is essential for stabilizing the elbow joint. Its reconstruction is recommended in both acute and chronic injuries in order to restore elbow stability and prevent early degenerative changes. Jiang et al. reported the case of a 13-year-old boy with chronic postero-lateral dislocation of the left elbow due to the absence of the coronoid process of the ulna. They reconstructed the coronoid process with the tip of the olecranon and achieved good stability of the elbow joint at a 2-year follow-up. This clinical case demonstrates that the reconstruction of the coronoid process of the ulna with the proximal end of the olecranon provides good mid-term results in children with elbow instability due to the absence of the coronoid process of the ulna.

De Maio et al. performed a systematic review of the literature to identify the best operative treatment for children with displaced olecranon fractures with or without associated injuries. They found that surgically treated fractures generally have a good prognosis and that tension band suture is the preferred fixation, although it is not recommended in older children due to the high risk of fixation failure. They also reported that the outcome was worse in patients with associated injuries.

The presentation, management, and evolution of fractures of the distal end of the humerus in children are complex and require special attention. With this in mind, the articles in the Special Issue *Elbow Injury in Pediatric Patients* offer valuable insight into the diagnosis and treatment of such conditions.
